# Robotic parastomal hernia repair with reinforced tissue matrix; lessons learned from our 74-patient cohort

**DOI:** 10.3389/jaws.2026.16508

**Published:** 2026-05-08

**Authors:** J. E. Baart, T. C. van Smaalen, A. L. A. Bloemendaal

**Affiliations:** Department of Surgery, Reinier de Graaf Gasthuis, Delft, Netherlands

**Keywords:** parastomal hernia repair, Pauli repair, reinforced tissue matrix, retromuscular, robotic

## Abstract

**Introduction:**

Patients with a stoma often develop a parastomal hernia (PSH). Treatment of PSH is challenging and results are often disappointing, with many patients developing multiple recurrences. In this study we aim to describe our experiences with the treatment of PSH using our robot assisted operative technique, by exploring patient history and PSH repair outcome and giving in-depth description of failures and successes. We try to identify and better understand predicting factors for failure, postoperative complications and recurrence in an attempt to make a small step towards a more patient-tailored approach to parastomal hernia repair.

**Methods:**

All (robotic) PSH repairs performed in our centre from March 2022 to January 2026 were registered in a prospectively collected database.

**Results:**

In this period 74 patients underwent a robotic PSH repair. Almost half of these patients (46%) had undergone one or more previous attempts to PSH repair. Nine recurrences occurred (12%). Complication rate was 31% (9.5% severe complications). IPOM mesh *in situ*, ileostomy and amount of previous repair attempts may be related to recurrence and post-operative complications.

**Conclusion:**

This study highlights the complexity for PSH repair, becoming increasingly more complex in recurrent cases. More (comparative) research is needed to establish a more patient-tailored approach to PSH repair.

## Introduction

Patients with a stoma will often develop a parastomal hernia (PSH), leading to a significant decrease in quality of life (QoL) on top of the loss of QoL due to the stoma itself [1, 2]. Treatment of PSH is a challenge and is considered to be a hazardous and often unsuccessful undertaking [3]. Whether results are even more disappointing in treatment of recurrent PSH cases is unclear, but this is highly probable as results in recurrent incisional hernia repair are worse than primary incisional hernia repairs [4]. A recent publication showed a 31% re-recurrence after repair of recurrent PSH [5].

In recent years several new techniques have been introduced, leading to a variety of operations being performed [6]. The gold standard is probably still the laparoscopic (modified) Sugarbaker technique [7]. The modification of this technique to a retromuscular mesh placement, the Pauli repair [8], is increasingly practised. A specially modified “funnelmesh” adaptation also shows promising results [9], as does the “sandwich-technique” [10].

In 2023 we reported on our early results and the lessons learned in our robotic approach in the treatment of PSH in 11 patients, using a modified robotic Pauli technique with a reinforced tissue matrix as mesh [11]. In this paper we will describe our ongoing learning in a 74-patient robotic PSH repair cohort with this mesh, consisting of all types of PSH and explore the differences and difficulties we have discovered along the way. We will explore the relation between patient characteristics, previous operations and type of stoma to assess success and failure of this technique in different PSH repair settings in an attempt to make a small (first) step towards more patient tailored surgery in PSH repair and to identify risk factors for post-operative complication and recurrence. Due to the retrospective nature of this analysis, with significant heterogeneity in patients and hernia characteristics, combined with small numbers and only 15-month median follow-up, we remain mainly descriptive in our report, but will explore relationship between complexity of repair and recurrent PSH repair using univariate analyses.

## Materials and methods

### Setting, patient selection and data collection

The study was conducted in the Reinier de Graaf Gasthuis (RdGG), a district teaching hospital with expertise in parastomal hernia repair surgery. All PSH repairs performed in our centre are registered in a prospectively collected database. Patients undergoing robotic PSH repair from March 2022 to January 2026 were included in this study. In all cases informed consent for use of information was obtained in the electronic patient file. All operations were performed using a Da Vinci Xi system (Intuïtive Surg, Sunnyvale, USA). The following data was collected: age at time of operation, gender, medical and surgical history, type of stoma, parastomal hernia classification according to the European Hernia Society [10, 12], American Society of Anesthesiologists (ASA) classification of physical status, Body Mass Index, surgical time, length of stay, post-operative 90-day complications, recurrence status and reoperation.

### Surgical indication

The decision to perform robot assisted PSH repair was multi-factorial and based on patient characteristics, surgical history and previous repairs, patient condition and patient preference and expectations. The option of reversal of the stoma was always considered first. Patients who were not suited for an extensive repair, e.g., due to frailty, were counselled for local procedures or a primary repair. Emergency cases were always performed by local repair also. Patients were counselled on the use of a non-synthetic mesh.

Whenever possible and necessary, patients were prehabilitated. Three important reasons for prehabilitation were pulmonary function, cardiac comorbidity and obesity. Patients were referred to a pulmonologist for pulmonary prehabilitation and underwent cardiac review to estimate operative risk and if possible, to adjust medication to improve cardiac function. A BMI of <33 was strived for and patients with higher BMI were referred for bariatric review or offered outpatient dietician of physiotherapy coaching to lose weight.

Patients were counselled that the primary choice of operation was a robotic Pauli repair with chance of conversion to robotic Sugarbaker or robotic sandwich repair if necessary. Conversion to an open repair was also mentioned for consent.

### Surgical procedure

The robotic Pauli technique was described in detail previously [9, 11]. In summary, all procedures were performed under general anaesthesia after intrathecal morphine was administered (necessitating an indwelling urinary catheter). Antibiotic prophylaxis was administered half an hour before the operation. Patients were placed in supine position, slightly tilted to the side contralateral to the stoma. Insufflation to 12mmHg was achieved by Veress needle to the left upper abdomen (Palmer’s point). Three robotic 8 mm trocars were inserted into the contralateral flank (opposing stoma). Adhesiolysis was performed as necessary. The stoma conduit was mobilized as much as possible to achieve a “free hanging” conduit with limited bulk in the subcutis. The retromuscular plane was developed around the stoma and a limited posterior component separation (PCS/TAR) was performed to further develop the plane deep into the flank. Approximation of the anterior fascia (around the conduit) was performed with 0 v-loc [Medtronic, Fridley USA]. An Ovitex mesh [TelaBio, Malvern USA] was placed and fixed to the lateral abdominal wall with v-loc 3/0 sutures [Medtronic, Fridley USA]. In most cases an Ovitex 1s permanent mesh (with 12 g/m^2^ polypropylene suture reinforcement) was used, which can be introduced down a 15 mm trocar, placed through the rectus muscle in the upper abdomen (either left or right). Mesh size was adjusted as necessary. Peritoneum/posterior layer was closed using v-loc 3/0 sutures [Medtronic, Fridley USA].

The surgical procedure has been altered several times in order to improve quality (or speed) of the operation. We describe a number of these technical adjustments here (some already previously described):

1. Additional suture fixation of the stoma conduit to the lateral abdominal wall, to mesh and peritoneum to reduce the chance of a sliding hernia recurrence
2. Explantation of old mesh was only be performed if necessary for a good redo-repair, because, in our experience, explantation of mesh is painful and increases the number of surgical complications (hematoma).
3. Fixation of Ovitex mesh to ensure proper ingrowth and to minimize dead space was increasingly deemed important. As visibility of the conduit is nil through this mesh we parachuted the mesh alongside the conduit with a running v-loc 3.0 [Medtronic, Fridley USA] to ensure proper tunnelling of the conduit behind the mesh.
4. The use of Ovitex mesh is often accompanied by the formation a relatively longstanding seroma but reduction of the early seroma has not led to a clear improvement of postoperative recovery in our cohort. We have not tried longstanding drainage due to perceived increased risk of secondary infection.
5. The use of glue to fixate the mesh was not successful for two reasons. Firstly, the application of glue must be a relatively quick and smooth effort, which, due to limited access and space in our set-up, is not straight forward. Secondly, the surface to which the glue is applied should be relatively clean and smooth, which is often not the case.

In a number of cases a robotic Pauli repair was not possible, either due to limited possibility to lateralize the conduit, or due to the absence of a (usable) posterior layer. In these cases, either a robotic Sugarbaker repair or a robotic sandwich repair was performed.

The robotic Sugarbaker is essentially the same procedure as the described robotic Pauli, but without closure of a posterior layer. An Ovitex 2S mesh was used in these cases to ensure a safe mesh surface for both stoma conduit and the other viscera.

The robotic sandwich repair was performed either intraperitoneally (in the absence of a posterior layer and the impossibility of adequate lateralization) or in retromuscular position. In both cases, a keyhole mesh was placed to reinforce the abdominal wall. A second mesh was placed in Sugarbaker configuration, but with limited lateralization.

### Follow-up

Standard follow up consisted of an in-office appointment at 8–12 weeks post-operatively, a telephone appointment after 6 months and again a yearly physical appointment after that. Additional imaging was performed only if indicated and therefor all recurrences were clinically diagnosed, followed by imaging if recurrence was suspected.

If patients presented with complications in their referring hospital, we were contacted by their local surgeons. Complications described are 90-day postoperative complications.

### Statistics

Data were analysed using IBM SPSS Software, PASW Statistics version 29.0.2.0. Descriptive statistics were used to present all data. Median values were presented with interquartile range 9IQR). Only factors that were expected to be relevant risk factors for adverse outcomes were provided with odds ratio’s and 95% confidence intervals (CI), using binary logistics. Categorical variables were analysed using Pearson Chi^2^, Fisher-Freeman-Halton Exact test. Continuous data were analysed for normalcy with Shapiro-Wilk and Q-Q plots to decide on (non)parametric tests: Mann Whitney U test was used for non-normal data. Median values were presented with interquartile range (IQR).

All analyses performed should be deemed explorative due to small number of event.

## Results

### Description of robotic cohort

**FIGURE 1:**
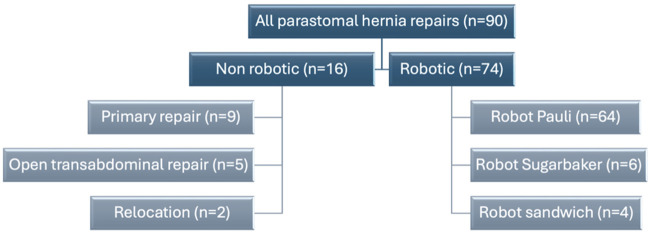
Flowchart of operations performed for parastomal hernia repair.

A total of 90 patients was operated for PSH between March 2022 and February 2026, of which 74 were treated robotically (baseline characteristics in Table 1). Sixteen patients underwent another type of repair (Figure 1) and are left out of the further description. In the robotic group, the Pauli technique was performed in 64 (86,4%) cases. Conversion to robotic Sugarbaker repair was necessary in 6 cases (8.1%), either due to the necessity of explantation of a synthetic (Sugarbaker) mesh, leading to obliteration of the posterior layer, or (in two cases) no utilisable posterior layer was present at all. Conversion to a robotic sandwich repair was performed in 4 cases (5.4%): 1 end ileostomy and 3 urinary conduit PSH repairs. In these 4 cases lateralization of the conduit was severely hampered by a short mesentery/ureter.

**TABLE 1:** Baseline characteristics of patients undergoing robotic parastomal hernia repair.

Characteristic	​	n	Range	%
Patient	Age (median)	68	37–89	​
	BMI (median)	29	22–41	​
	Female (n)	39	​	53%
Stoma type	Colostomy	45	​	61%
	Ileostomy	15	​	20%
	Urinary conduit	14	​	19%
EHS classification	I	20	​	27%
	II	8	​	11%
	III	32	​	43%
	IV	14	​	19%
Recurrent PSH	no	40	​	54%
	Yes	34	​	46%
Number of previous PSH repairs	1	13	​	18%
	2	12	​	16%
	3	3	​	4%
	4	4	​	5%
	*5*	*0*	​	​
	6	1	​	1%
	7	1	​	1%
Patients with mesh *in situ*	no	47	​	64%
	Yes	27	​	36%

In 22 cases (30%) a concomitant (midline) incisional hernia was present (EHS classification II & IV). In these cases, the midline hernia was addressed by a retromuscular repair and a synthetic mesh (Progrip; Medtronic, Fridley, USA) was placed separate to the Ovitex mesh used for the PSH. In cases where the synthetic mesh would overlap the Ovitex mesh for adequate overlap, the synthetic mesh was placed onto the Ovitex mesh, to avoid any contact between the synthetic mesh and the stoma conduit.

### Complications and mortality

Complications arose in 23 patients (31,1%), of which 7 (9.5%) were severe complications (Clavien Dindo ≥ 3) and are summarized in Table 2. Further descriptive analytics are given in Table 3.

**TABLE 2:** Complications in total cohort.

Clavien Dindo	n (%)	Complication	n
0	51 (69%)	​	​
1	7 (9.4%)	Seroma	2
		Hematoma (SSO)	3
		Prolonged pain	1
		CVC infection	1
2	9 (12.2%)	Ileus	3
		Infected hematoma (SSI)	1
		Urinary tract infection	2
		Pneumonia	2
		Pulmonary embolism	1
3	6 (8.1%)	Infected hematoma (SSOPI)	1
		Conduit ischemia	2
		Incarcerated recurrence	1
		Perforation bowel	1
		Ureteric injury	1
5	1 (1.4%)	Death	1

SSO, surgical site occurrence.

SSI, surgical site infection.

SSOPI, surgical site occurrence necessitating procedural intervention.

**TABLE 3:** Complications per patient or operative characteristic.

Characteristic		Complication (n)	%
All		23	31%
Colostomy		10	22%
Ileostomy		8	53%
Urinary conduit		5	36%
BMI	<25	3	38%
	25–30	12	38%
	30–35	5	26%
	35–40	2	15%
	>40	1	50%
Previous PSH repairs	0	10	25%
	1	0	0%
	2	5	42%
	>2	8	89%
Type of repair	Pauli	17	27%
	Sugarbaker	2	33%
	Sandwich	3	75%
PSH repair mesh *in situ*	no	12	26%
	IPOM	8	62%
	RM	2	18%

Complications arose in 53.3% in end ileostomies compared to 22.2% in colostomies and 35.7% in urinary conduits. In patients with ≥2 previous repairs, complications occurred in 53.8% compared to 28.1% in patient who underwent no or 1 previous repair.

A univariate analysis of complications related to type of stoma, number of previous repairs and whether previous mesh was still *in situ* during our operation is shown in Table 4.

**TABLE 4:** Univariate analysis of complications related to type of stoma, number of previous PSH repairs and *in situ* mesh.

Characteristic	No complication (n = 51)	Complication (n = 23)	OR	95% CI	p-value
Type of stoma (ref: end colostomy, %)					
Urinary conduit	9 (64.3)	5 (35.7)	1.944	0.530–7.132	0.316
End ileostomy	7 (46.7)	8 (5.33)	4	1.164–13.740	0.028
Previous repair (ref: 0, %)					
1	13 (100)	0	0	0	0.999
≥2	7 (36.8)	12 (63.2)	5.143	1.588–16.657	0.006
Mesh *in situ* (%)	16 (59.3)	11 (40.7)	1.948	0.709–5.354	0.196

### Severe complications

One patient died after a robotic Pauli repair. His body mass index was 41 kg/m^2^ and he had a longstanding PSH with significant loss of domain necessitating an open reduction of the hernia contents through a peristomal incision. There was a bowel injury, which was not recognized until day 3 after surgery. He was reoperated and a short segment of small bowel was removed. Thereafter, the patient rapidly developed multiorgan failure and died within 24 h.

One patient developed a hematoma in the subcutaneous cavity after robotic Pauli, which subsequently infected. The extra-abdominal conduit became necrotic and perforated on day 5. A laparotomy was performed, where no important intraabdominal complications were seen. Notably, the intraabdominal conduit was unaffected, and the mesh was not visibly contaminated. Nevertheless, the mesh was explanted, and a new stoma was formed on the contralateral side of the abdomen. The patient developed an incisional hernia in the old stoma port site and a parastomal hernia at the new location at 4 months follow-up.

One patient underwent PSH repair for a previously relocated urinary conduit. The initial formation of the conduit was complicated by an infection, leading to resection of the conduit and left nephrectomy. A new conduit was formed in the left abdomen. The single (right) ureter now transversed the abdomen over the bowels from right to left severely hampering lateralisation of the conduit. After robotic sandwich repair, urine production declined over the first postoperative day and on day 2 a CT scan was performed, showing a urinoma proximal to the kidney. We assume that, in attempting to achieve some lateralization of the conduit, a traction lesion of the proximal ureter arose, which was unrecognized during the operation. The patient received a nephrostomy catheter, and a stent was placed in the ureter. Patient recovered without lasting complications. The stent and nephrostomy were removed 6 weeks later and no recurrence of PSH has arisen.

One patient, in which we removed the intraperitoneal mesh (after laparoscopic Sugarbaker repair) and performed a robotic Sugarbaker with reinforced tissue matrix developed an infected hematoma, which was drained percutaneously. Over time the infection did not completely subside, leaving the patient with a chronic abscess between mesh and abdominal wall. The reason for stoma formation in the first place had been a severe slow-transit colon. We performed a laparotomy with subtotal colectomy with ileo-sigmoidal anastomosis, with an uneventful course.

One patient, who previously underwent a Hartmann’s procedure for diverticular perforation, developed a perforation in the subcutaneous conduit (which still contained many diverticula) necessitating a relaparotomy, resection of all affected colon and relocation of the stoma.

One patient developed ischemia of the stoma conduit, with no other complications. A relaparoscopy was initiated but converted to a laparotomy. The conduit was resected and a new colostomy was placed through the (unaffected) mesh, which was left *in situ* in keyhole configuration. The patient developed a wound infection in de midline laparotomy which was treated conservatively.

### Recurrence after robotic PSH repair

Nine recurrences arose in our robotic PSH repair cohort (12%) at median 15-month follow-up (Table 5). Eight recurrences developed after robotic Pauli repair (12.5%), one after robotic Sugarbaker repair (20%) and none after a sandwich repair. Median time to recurrence was 4 months (range 2–12 months; IQR 2 months). All recurrences arose within the first year after surgery.

**TABLE 5:** Recurrence rate per characteristic.

Characteristic		n	%
All		9	12%
Colostomy		7	16%
Ileostomy		2	13%
Urinary conduit		0	0%
BMI	<25	1	13%
	25–30	3	9%
	30–35	3	16%
	35–40	2	15%
	>40	0	0%
Previous PSH repairs	0	3	8%
	1	0	0%
	2	3	25%
	>2	3	33%
Type of repair	Pauli	8	13%
	Sugarbaker	1	20%
	Sandwich	0	0%
PSH repair mesh *in situ*	no	2	4%
	IPOM	4	31%
	RM	2	18%
All		9	12%
No complication		5	10%
Clavien dindo 1/2		3	19%
Clavien dindo 3/5		1	14%

Three recurrences arose in the 40 patients who were not previously operated for PSH (7.5%), of which two were sliding of the conduit. In cases with only one previous attempt to repair (n = 13) no recurrence was seen. Conversely, looking at multiply recurrent cases (two or more recurrences) the number of further recurrences after our surgery increased to 6 out of 21 cases (28.6%).

Twenty-four patients had previously placed mesh (for PSH repair) still *in situ* (intraperitoneal or retromuscular) of which 6 (25%) developed a re-recurrence after our attempt for recurrent PSH repair. In the intraperitoneal mesh group 4 out of 13 developed a recurrent PSH (30.8%), while in the retromuscular mesh group 2 out of 11 patients developed a recurrence (18.1%).

A univariate analysis of recurrence related to type of stoma, number of previous repairs and whether previous mesh was still *in situ* during our operation is shown in Table 6.

**TABLE 6:** Univariate analysis of recurrence related to type of stoma, number of previous PSH repairs and *in situ* mesh.

Variable	No recurrence (n = 65)	Recurrence (n = 9)	OR	95% CI	p-value
Type of stoma (reference: end colostomy, %)					
*Urinary conduit*	14 (100)	0	0	0.00–0.00	0.999
*End ileostomy*	13 (86.7)	2 (13.3)	0.835	0.154–4.540	0.835
Previous repair (reference: 0, %))					
*1*	13 (100)	0	0	0.00–0.00	0.999
*>1*	14 (73.7)	5 (26.3)	4.405	0.928–20.917	0.062
Mesh *in situ* (%)	20 (74.1)	7 (25.9)	7.7	1.467–40.415	0.016

### Follow-up surgery after recurrence

Of the 9 recurrences, 4 developed a sliding (hernia) of the stoma conduit, of which three underwent a local repair to shorten the conduit. So far, these patients have had no new complaints. One patient was not significantly troubled by the recurrent bulge and has not undergone a second intervention.

Five patients developed a new true parastomal hernia necessitating further operations. Two of these patients underwent a local repair with reduction of the herniating abdominal content and refashioning of the mesh, which had been dislocated from the abdominal wall. Both these patients developed a further recurrence. One underwent an open operation with relocation of the stoma and placement of a Sugarbaker configuration mesh (reinforced tissue matrix). The other awaits further (open) surgery. One patient underwent an open bilateral PCS/TAR (posterior component separation/transversus abdominis release) with a large synthetic mesh and placement of a new reinforced tissue matrix to separate the stoma conduit from the synthetic mesh. This reintervention has been successful to date. One patient developed an early incarcerated recurrence (6 weeks after robotic Pauli repair) and first underwent a local repair to treat the incarceration, rapidly followed by a non-incarcerated recurrence, after which we performed an open relocation of the stoma. She has developed a new PSH at the new site but currently has no wish for repair. The last patient has undergone a new attempt at a robotic (sandwich) repair of his (now 8th) recurrence of PSH.

### Mesh infection

No mesh infections were seen in our cohort. In 3 cases the mesh was removed during a reoperation for a severe complication. In one case this removal was due to a chronic abscess, which may have been related to mesh. During this operation the mesh seemed not to be affected, but this is of course not evincible. No mesh erosions were seen.

## Discussion

In this paper we describe the results of our cohort of 74 patients operated robotically for a parastomal hernia using a reinforced tissue matrix as mesh. To date most (retrospective) studies on PSH repair contain either a mix of different operative techniques, without clear mention of the reason for choice of technique or contain one technique in a relatively small number of patients, often with a high variability in patient characteristics and history. Although current study describes the findings in a relatively large cohort when compared to other papers on this subject, the small number of cases and significant heterogeneity in patients, still limits the generalisability of our findings.

We report a recurrence rate of 12% in our cohort. The relatively high recurrence rate, when compared to a Norwegian study reporting on 56 retromuscular mesh repairs (Pauli repairs) with 5.6% recurrence [13], may be explained by a high number of recurrent cases in our cohort (46%), of which the majority was multiply recurrent (28% of total cohort), whereas the Norwegian cohort had 25% recurrent cases, of which 5 patients had multiple repairs (9% of total cohort).

Patients having undergone 2 or more previous attempts at repair were more at risk of developing a recurrence after our repair, which is in line with a Spanish nationwide registry analysis on recurrent incisional hernia repairs [4], but contradicted by others [14, 15].

Szczepkowski et al. developed the Hybrid Parastomal Endoscopic Repair (HyPER) technique, which combines laparoscopic and open approaches. They report an astonishing recurrence rate of 5.5% on a cohort of 200 patients consisting of mainly EHS Type III or IV hernias with a follow up range of 2–6 years [12, 16].

Of course, the choice of mesh may influence recurrence. The use of a permanent synthetic mesh is still the “gold standard” in PSH repair. We are very clear to our patients that we deviate from this standard and explain our choice for the Ovitex mesh in all cases. For incisional hernia repair the Ovitex (reinforced tissue matrix) mesh has shown promising result [17]. In the BRAVO study, the OviTex 1S was shown to be non-inferior regarding recurrence rates after ventral hernia repair at 24-month follow up, compared to synthetic mesh. But whether these results are extrapolatable to parastomal hernia repair is unclear [18]. Our rationale for choosing a synthetically reinforced biological mesh has been previously described and is largely due to the rare but devastating complications of erosion and infection of synthetic mesh use in PSH repair [19, 20]. A common misconception is that Ovitex biological meshes will be fully absorbed by the body, thus compromising endurance of the PSH repair. Reinforced biological meshes are designed with a biologic base to facilitate tissue remodelling, promoting integration of the mesh into host tissue, and contain a low synthetic polymer footprint for reinforcement as shown in a case report reporting on a very complex open abdomen treatment [21].

In our cohort 31% of patients suffered a complication, of which the majority (70%) was Clavien Dindo type I and II. Severe complications arose in 9.5% of the total group, which is a high number. However, considering the complexity of the cohort this, in our opinion, is expected and acceptable. The higher number of complications in ileostomies compared to colostomies and urinary conduits could be related to the presence of IBD, as shown in a comprehensive review [22]. End ileostomies were mainly fashioned after (procto)colectomy for inflammatory bowel disease. In these procedures a surgeon will often chose to perform a “close colon” resection, leading the bulk of the right mesocolon *in situ*. During formation of the ileostomy this leads to a bulky mesentery alongside the stoma conduit, containing the ileocolic vasculature. This, in our experience leads to two problems:

1. Bulk. The ileocolic mesocolon is bulky and may push the mesh off the abdominal wall as described by Muysoms et al. [23].
2. Adhesion. The mesocolon can be strongly adherent within the subcutis, making it more difficult to achieve full adhesiolysis of the subcutis, which we believe to be very important for successful PSH repair.

One may argue that the use of a minimally invasive (robotic) approach could lead to an increase in perioperative complications due to limited working space and the absence of haptic feedback in a often challenging operative environment. In general, surgeons will convert to open surgery, when running into difficulty in adhesiolysis in a minimally invasive operation. PSH surgery, especially in the presence of previous mesh, often encompasses a challenging adhesiolysis. In our series, two patients suffered a bowel perforation, one peroperatively and 1 days after the operation. In one patient the perforation was in the subcutaneous stomaconduit, which had multiple diverticula. The perforation arose days after the operation but was probably due to the adhesiolysis of the hernia sack. This adhesiolysis is definitely more challenging in minimally invasive surgery compared to open. For this reason we now more often perform an open adhesiolysis of the hernia through a small parastomal incision, when we cannot clearly and safely perform an adequate adhesiolysis robotically. After adhesiolysis and reduction of hernia content we close the incision to proceed with the robotic procedure. We performed this technique in the second patient suffering a bowel perforation. We think the bowel perforation was probably made during open adhesiolysis in the hernia, which was very challenging in a long standing, loss-of-domain PSH. The perforation was not recognized during the operation. Patients’ high BMI and considerable loss-of-domain led to a limited view intraabdominally, which severely hampered the robotic procedure. It could be argued that this case should not have been performed robotically, although the added morbidity of an open operation was also unfavourable.

Over 40% of patients with a stoma will develop a symptomatic PSH, but only a small minority of these patients will undergo surgery. In Denmark, counting over 6.000.000 inhabitants, only 409 patients underwent a form of PSH in a 3-year period. This was already an improvement after centralization of complex hernia care [24]. Furthermore, when a patient does undergo surgery, even in a high-volume hernia centre, often a variety of techniques is used, with a highly variable success rate [25]. Looking at success rates of different mainstream techniques Rautio et al. show quite comparable recurrence and complication rates in a nationwide cohort study [6]. The question remains, why so few patients are operated and with such a variety of techniques? Will further development of new techniques and further specialization of surgeons increase the number of treated patients and lead to a more patient-tailored approach? And will this lead to a significant improvement in quality of life for patients living with a PSH in general?

Interestingly, papers reporting on these different surgical PSH-treatments usually only make a mere mention of the number of recurrent cases having been treated. The type of stoma is mentioned, along with other patient related characteristics. But, seemingly, the treatment of the PSH is often considered a “one-size-fits-all” undertaking. Should a multiply recurrent parastomal hernia of a urinary conduit be considered comparable to a first repair of an end colostomy? Should we not be tailoring the repair of hernias to the type of stoma, the number of recurrences, previous operations and defined patient characteristics, instead of trying to find the one operation, that will treat all PSHs?

One might be critical about our decision to perform parastomal hernia repair in patients with a BMI over 35 kg/m^2^ because of the known increased complication rate in this subgroup [26]. In these cases, bariatric surgery or other forms of prehabilitation had either failed or was not possible at the time. With these patients, thorough pre-operative counselling was performed to weigh possible benefits and adverse outcomes of surgery as compared to not performing a surgical intervention. Patients with an increased operative risk who decided to undergo surgery were usually experiencing a high burden of symptoms such as functional problems with the ostomy, pain, or incarceration. Also, because we are a national referral centre for parastomal hernia repair, patients often had a persisting wish for surgical correction and had already undergone extensive counselling in their referring centre before they were referred to our centre. Of course, some of our patients decided against parastomal hernia repair after shared decision making, but these patients were not included in our study.

There are important limitations to this study, which strongly affect the generalizability of the findings and the transferability to other institutions.

1. This paper reports on patients operated in a specialized, tertiary referral centre for abdominal wall surgery, with a high level of access to the robotic platform.
2. As in most reports on parastomal hernia, we report on a small and strongly heterogenous group of patients, limiting generalizability of the results.
3. Another definite limitation is the relatively short follow-up time. The number of patients operated in our centre, using our robotic technique, has increased significantly over the years with the bulk of the patients operated in the past 2 years.
4. Our standard follow-up procedure does not include standard imaging to rule out (radiological) recurrence. This may lead to an underestimation of the true number of recurrences.
5. We did not collect quality of life data and cannot present the rates of post-operative (chronic) pain. This, for us, is a highly important limitation of our study. We strongly believe that surgery for PSH is a “quality of life-procedure”. More than radiological recurrence, so important and emphasized in oncological surgery, patients should be operated to improve their daily quality of life. To a patient, a radiological recurrence is of minimal importance as long as the quality of life remains improved.
6. We cannot draw independent predictors from our study results. However, we did find that multiply recurrent hernia was associated with adverse outcomes.
7. This is a descriptive report on the outcomes of our robotic technique. We have not compared our group to a historical cohort or a concurrent group of patients operated with another technique. This impedes the possibility of making a substantiated conclusion on the benefits of our (robotic) technique compared to open or laparoscopic techniques.
8. This study describes the development of a new technique from the very first operation, therefor including the learning curve and the adjustments/improvements in technique made over time, which, combined with short follow-up and a strongly heterogenous patient population, limits the possibility of creating a standard operations protocol from these results, which strongly limits the reproducibility of the technique based on this paper alone.

In this paper, we describe our ongoing learning curve with parastomal hernia repair and make a first attempt at determining factors related to recurrence or complication, although our small subgroup sizes and heterogenicity of patient’s severely limit analytical power. However, because not much literature is available, we believe our first attempt at finding predictors for recurrence after PSH repair is beneficial for the field. In a later stadium, based on current and future findings, specific research can be performed to further tailor surgical decision making for specific cases and the possibility of creating a standardised decision -making protocol.

In conclusion, the results of our robotic repair for parastomal hernia using a reinforced tissue matrix seem encouraging, even in a very challenging cohort of patients, often having undergone multiple previous attempts to a PSH repair. However, as this is a descriptive study, without a direct or indirect comparative analysis to other surgical approaches, any judgement or statement on superiority of current technique is incorrect. We believe further (collective) comparative studies are needed to further specify which surgical technique should be performed towards a more patient-tailored approach to PSH repair.

## Data Availability

The raw data supporting the conclusions of this article will be made available by the authors, without undue reservation.
